# Can We Accurately Measure Axial Segment Coordination during Turning Using Inertial Measurement Units (IMUs)?

**DOI:** 10.3390/s20092518

**Published:** 2020-04-29

**Authors:** Fuengfa Khobkhun, Mark A. Hollands, Jim Richards, Amornpan Ajjimaporn

**Affiliations:** 1 College of Sports Science and Technology, Mahidol University, Salaya, NaKhon Pathom 73170, Thailand; fuengfa.kho@mahidol.edu; 2 Brain and Behaviour Lab, Research Institute for Sport and Exercise Sciences, Liverpool John Moores University, Tom Reilly Building, Byrom Street, Liverpool L3 3AF, UK; m.a.hollands@ljmu.ac.uk; 3 Department of Physical Therapy, Faculty of Physical Therapy, Mahidol University, Salaya, NaKhon Pathom 73170, Thailand; 4 Allied Health Research Unit, School of Sport and Health Sciences, University of Central Lancashire, Preston PR1 2HE, UK; JRichards@uclan.ac.uk

**Keywords:** inertial measurement unit, turning, kinematics, whole-body coordination, Vicon

## Abstract

Camera-based 3D motion analysis systems are considered to be the gold standard for movement analysis. However, using such equipment in a clinical setting is prohibitive due to the expense and time-consuming nature of data collection and analysis. Therefore, Inertial Measurement Units (IMUs) have been suggested as an alternative to measure movement in clinical settings. One area which is both important and challenging is the assessment of turning kinematics in individuals with movement disorders. This study aimed to validate the use of IMUs in the measurement of turning kinematics in healthy adults compared to a camera-based 3D motion analysis system. Data were collected from twelve participants using a Vicon motion analysis system which were compared with data from four IMUs placed on the forehead, middle thorax, and feet in order to determine accuracy and reliability. The results demonstrated that the IMU sensors produced reliable kinematic measures and showed excellent reliability (ICCs 0.80–0.98) and no significant differences were seen in paired *t*-tests in all parameters when comparing the two systems. This suggests that the IMU sensors provide a viable alternative to camera-based motion capture that could be used in isolation to gather data from individuals with movement disorders in clinical settings and real-life situations.

## 1. Introduction

Three-dimensional motion analysis is one of the most important investigative methods to study human locomotion, which includes movements such as changing direction and turning on the spot [1]. In both clinical and research settings, objective kinematic measurements of body movements are needed to identify impairments and to evaluate the effects of therapeutic interventions. Investigating abnormal movement patterns provides useful information that can aid clinical decision making. This information also plays a role during the follow-up stage for patients by helping to determine the effectiveness of a particular exercise or treatment intervention [2]. For example, there is a consensus among physiotherapists that performing movement analysis is beneficial for rehabilitation in individuals with Parkinson’s disease; this includes the analysis of functional mobility, pre- and post-rehabilitation planning and follow-up assessments [3].

Camera-based 3D motion analysis systems, such as Vicon and Qualisys to name just two, are considered to be the gold standard laboratory tool for the analysis of human movements which have been shown to have a high degree of accuracy [3,4,5,6]. However, the cameras, force platforms and software programs required are expensive, time-consuming and require skilled technicians and a large calibrated measurement volume. Therefore, using such equipment in a clinical setting is prohibitive. It is also not clear to what extent data collected in a laboratory environment are representative of natural performance in daily life [7]. In recent years, Inertial Measurement Units (IMUs) have been shown to be a viable alternative monitoring solution to assess movement in clinical and research settings [8,9]. Furthermore, the validation of biomechanical parameters in sport using IMU systems has provided useful comparisons with optical camera systems [10,11].

These sensors are housed in small boxes that can be attached to different body segments and provide linear acceleration and Euler angle measurements. They also have low power requirements and allow continuous measurements outside of the laboratory environment, and so can be used in real-life contexts [3,11].

The measurement of movements other than level walking are key in the assessment of individuals with neurological impairments. One such movement is turning, which is frequently used as part of routine clinical assessments and includes “turn 180˚” and “turn 360˚”, which have been used in the assessment of falls risk in the elderly [12], and movement disorders including Stroke Survivors [13] and in people with Parkinson’s Disease [14]. Therefore, the aim of this study is to validate the use of IMUs to examine turning characteristics in healthy participants by comparing IMU data to data generated by a Vicon motion analysis system. The results will allow us to explore whether IMU devices could possibly be used in isolation to augment turning movement assessments in individuals with neurological conditions within clinical settings and real-life situations.

## 2. Materials and Methods

### 2.1. Participants

Twelve healthy adults (six males and six females, mean age 23.58 ± 3.15 SD years, mean weight 63.55 ± 10.56 SD kilograms, and mean height 165.97 ± 9.16 SD centimeters) participated in the study. All the participants were asked to read a participant information sheet and sign an informed consent form approved by Liverpool John Moores Research Ethics Committee (REC) (Reference no. 16/SPS/001). Participants were asked to wear a sleeveless shirt and close-fitting trousers. 

### 2.2. Experiment Procedure

Participants stood approximately four metres in front of a projector screen (2.74 × 3.66 m, Cinefold Projection Sheet, Draper, Inc., Spiceland, IN, USA). Prior to each trial, a video was projected onto the screen showing an animation demonstrating the way we would like the participant to turn, in accordance with turning speed conditions. Participants were asked to turn at three speeds: fast (1.5 s), moderate (2 s) and slow (3 s), as previously suggested for turn 180˚ turning tasks [5]. Five trials were recorded for each turning condition and turns to the left or right sides were randomised, resulting in a total of fifteen trials for each participant.

### 2.3. Data Acquisition

**Figure 1:**
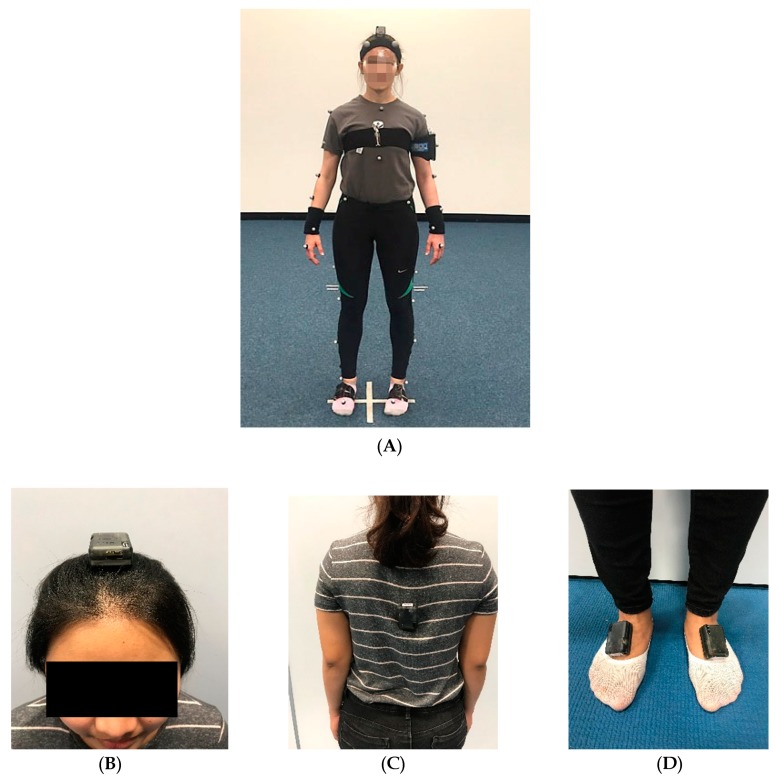
(**A**) Markers and inertial measurement units (IMUs) attached to the participant (**B**–**D**) IMUs attached to separate segments.

Thirty-nine reflective spherical markers were attached on the bony prominences of the participants and tracked using a ten camera Bonita motion analysis system (Vicon, Oxford, UK) at a sampling frequency of 200 Hz. The Plug-In-Gait model (Vicon^®^, 2002) was used to calculate joint kinematics, and anthropometric data were measured. In addition, four-IMU sensors size 33 × 42 × 10 mm (x-Inertial Measurement Units, x-io Technologies, LTD., UK) were attached to the following body segments: the centre of the forehead, middle thorax, the centre of the left and right foot (Figure 1). The IMU devices collected data at a sampling frequency of 256 Hz and the angular velocity of the body segments were recorded in real time. The IMU on-board sensors included tri-axial gyroscopes, accelerometers, and magnetometers. The IMUs featured a sensor fusion algorithm that used the on-board sensors to compute a measurement of orientation relative to the Earth. The algorithm can operate in the IMU or Attitude Heading Reference System (AHRS) mode, with the IMU mode only using the gyroscopes and accelerometers. The IMU mode provided an accurate measurement of the yaw, pitch and roll components of orientation but did not provide an absolute measurement of head orientation, whereas the AHRS mode used all of the on-board sensors, so that the orientation measurements were free from drift. Therefore, the AHRS mode provided estimates of the sensors’ orientation with respect to a global, fixed coordinate system and was therefore used in this current study. The IMU data were able to be viewed in real time, and were exported using an on-board SD card; this was then processed in MATLAB and Microsoft Excel.

### 2.4. Data Processing and Data Analysis

For data processing, the IMU data were re-sampled to 200 Hz, which corresponded to the sampling rate of the Vicon data. The two angular displacement time-series data streams were temporally aligned using a MATLAB (R2016b) programming environment that used the cross-correlation function (xcorr). This function calculated the time lag between the two data streams that corresponded to a maximum correlation coefficient. Further data processing was performed on the displacement, velocity, and acceleration profiles using the aligned data. This method was performed on the data from the Vicon system as a reference for validation, and the two data streams generated were synchronized using previously published research [15].

#### 2.4.1. Head and Thorax Data Processing

Segment angular displacement and angular velocity profiles were then compared from the Vicon and the IMU data. The head and thorax data from the two datasets were then filtered using a dual pass low-pass 4th-order Butterworth filter using a cut-off frequency of 6Hz [15]. Segment angular displacement data were then differentiated to yield angular velocity profiles for each segment.

#### 2.4.2. Step Analysis

The foot data were passed through a dual low-pass 4th-order Butterworth filter using a cut-off frequency of 10 Hz, which provided a clear event marker for each step. The step events were defined as the positive zero crossing preceding and the negative zero crossing following, a velocity which surpassed a threshold of 15% of the maximum step velocity. Each step onset was then determined as the first frame of the step interval with a velocity greater than or equal to 30 deg/s. Following the peak velocity of the individual step, step end-time was defined as the first frame at which velocity fell below 30 deg/s [15]. Thereafter, individual step size, number of steps, step frequency and step duration were determined from the timing of step onset to step end. These criteria were used to determine the rotation onset time, end time, and all dependent variables for each segment and individual stepping characteristics using a previously published methodology [15].

### 2.5. Statistical Analysis 

All statistical analyses were performed in SPSS (version 23.0). Bland–Altman plots were used to describe the agreement between the two measurement techniques by constructing limits of agreement. These statistical limits were calculated using the mean and standard deviations (SD) of the differences between the Vicon and IMU data [16,17]. Intraclass correlation coefficients (ICC) were used as indices of reliability to estimate the population variances based on the variability between the Vicon and IMU data. The ICC demonstrates a value from zero (which implies no agreement) to one (which implies perfect agreement). Furthermore, paired *t*-tests were used to compare the means of all axial segment movement parameters and stepping parameters obtained using the two systems, which included: reorientation onset of head (1), thorax (2) and feet (3, 4), head and thorax end-time (5, 6), peak head yaw velocity (7), peak head–thorax separation angle (8), total step (9), turn duration (10), step frequency (11) and step size (12). All mean values are presented with SD unless stated otherwise. The Bonferroni correction was applied for the 12 *t*-tests, which resulted in a corrected alpha of *P* < 0.004. 

## 3. Results

**Figure 2:**
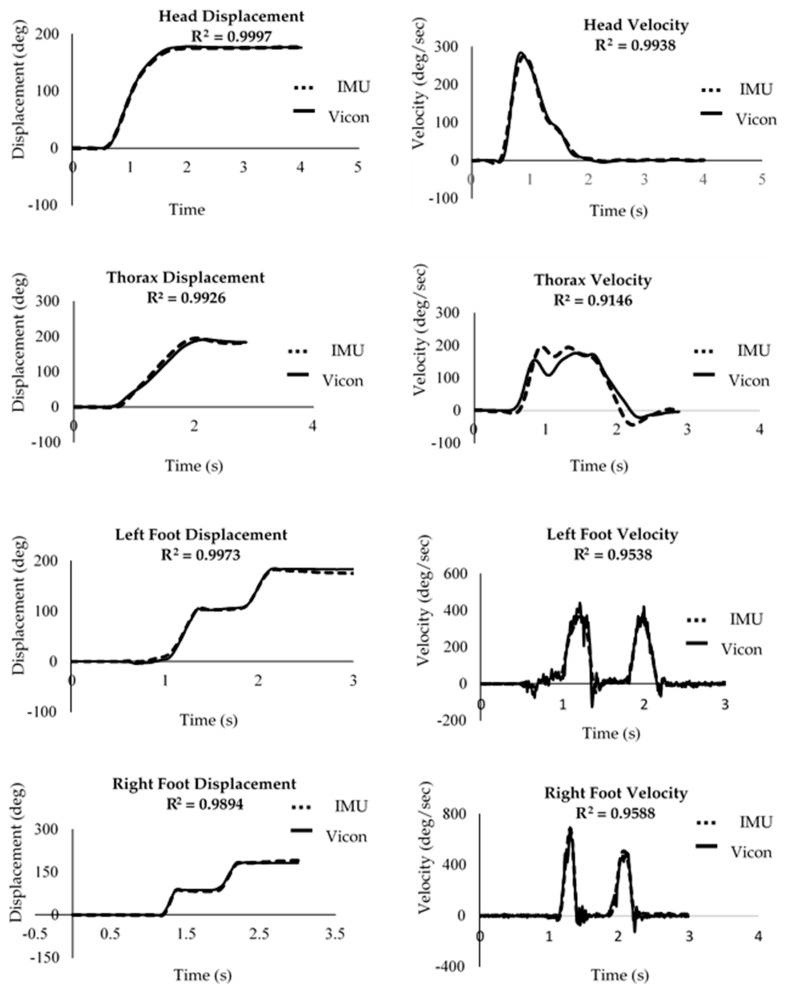
Examples of the angular displacement and velocity data collected during one trial. The solid lines represent data collected by the Vicon motion analysis system and the dotted lines the data collected by the IMU sensors.

An example of the raw angular displacement and velocity waveforms obtained during a turning trial at moderate speed when turning to the right is shown in Figure 2. The displacement and velocity time series of the head and the feet measured from the two systems closely resemble each other, but small differences were evident in the thorax plots. This observation was consistent for all trials under all speed conditions for all participants. The similarities between the waveforms measured from the Vicon motion analysis system and IMU data during the experiment in all speed conditions are shown in Table 1. In addition, the box and whisker plots of the R^2^ values and the linear equations y = mx + c are also presented. The present study focused on m (the gradient of the line) and c (the y intercept of the datasets with the ordinate axis). It is evident that the two systems measured similar displacement and velocity waveforms for the segments, and the two systems provided comparable data for all turning speed conditions.

**Figure 3:**
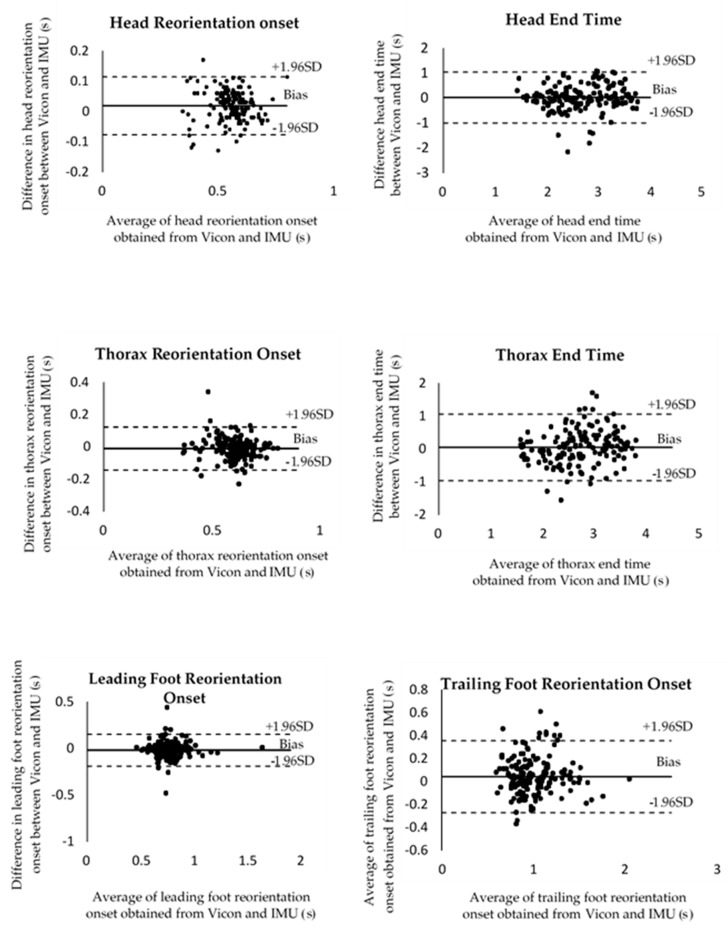

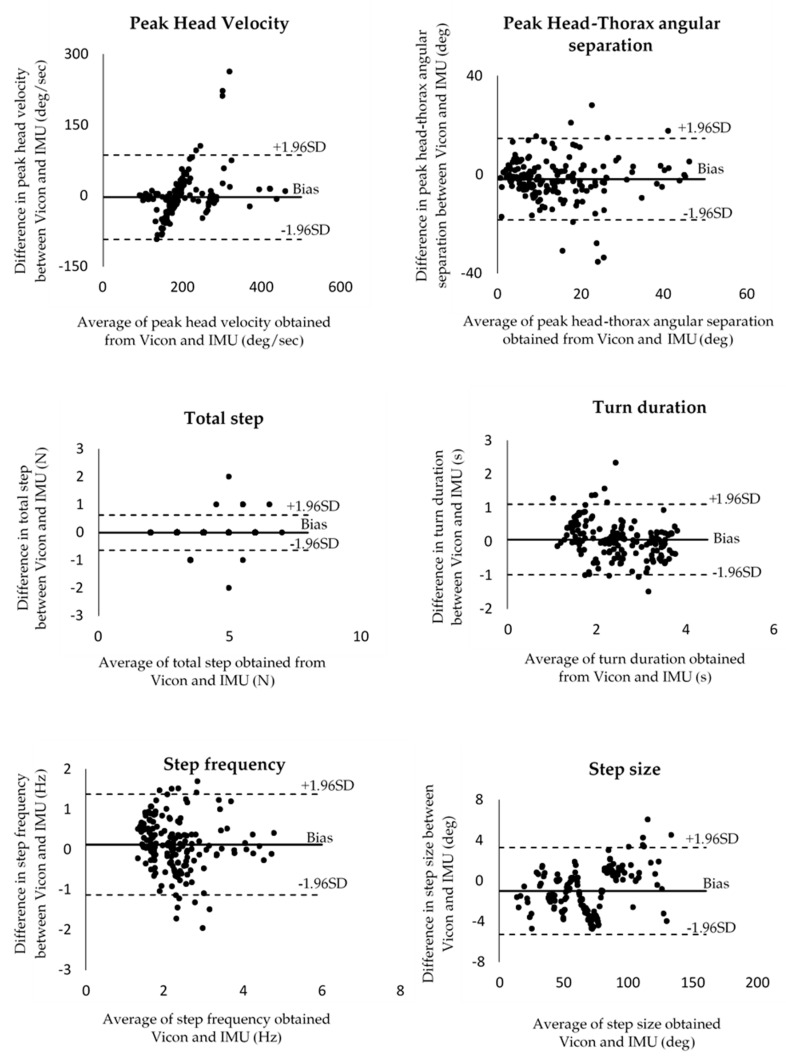
Bland–Altman plots for all variables. **Solid line** systematic bias (mean); **dash lines** limits of agreement.

Bland–Altman plots were used to show the assumptions of normality of differences and the limits of agreement, which were calculated using ±1.96 times the SD of the differences between the systems [14,15], and an ICC over 0.75 was considered ‘excellent’, and 0.4–0.75 as ‘fair to good’ [18]. The resulting graph in Figure 3 is a scatter plot XY graph in which the Y-axis shows the difference between the Vicon and IMU data, while the X-axis represents the average of these measures. Therefore, the difference between the measurements of the two data sets are plotted against the mean of the two measurements. All axial segment movement parameters showed no heteroscedasticity. Table 2 presents the averaged values from 180˚ trials from each variable for the two systems. The levels of agreement between the two systems were excellent for all variables (ICC between 0.80 and 0.98).

## 4. Discussion

The aim of this study was to validate the use of IMUs for the measurement of axial segmental coordination during turning in healthy participants by comparing data generated by a camera-based Vicon motion analysis system. The IMUs showed good agreement for the measurements of displacement and velocity of the head and the feet when compared to the Vicon system, which was demonstrated by very high ICC values during all three turning speeds.

The Bland–Altman plots demonstrated a narrow range of limits of agreement for all axial segment movement parameters. In addition, there was a small range of variability around the mean of the parameters, including head, thorax and feet reorientation onset and end time, peak head–thorax separation angle, turn duration and step frequency. It may be concluded from the results of the Bland–Altman plots and the high levels of agreement (ICCs between 0.80 and 0.98) that the two systems produce equivalent measurements of axial segment movement parameters during the 180-degree standing turn [7,17]. However, there was a small range of variability between the two systems. Several factors may have contributed to these results. First, the defined marker or position calculations between the two systems could explain the differences in displacement [19]. The Vicon motion analysis system recordings used reflective markers which were placed on the body segments of the subject on anatomical landmarks using the Plug-in-gait model. The 3D positions of these markers were recorded at 200 Hz using a ten-camera optical tracking system. Furthermore, the Plug-In-Gait model (Vicon^®^, 2002) was used to determine the angular displacement of the head, thorax and right feet in the global reference frame. With the IMU system, sensors were attached directly to the head and thorax as well as to the feet; this system incorporates algorithms that provide estimates of the sensor’s orientation with respect to a global, fixed coordinate system. This orientation can be represented by Euler angles in the local segment as well as by the estimates of sensor orientations, which require the use of magnetometer measurements with respect to a common global reference frame. Therefore, the difference between the data-processing methodologies of the two systems might have resulted in these differences in measures of displacement. Second, the variation in the position of the IMU sensors could explain the differences in the measured axial movement parameters; for example, the limitation of places to attach the IMU sensors on the thorax region meant that the IMUs had to be placed in a vertical orientation. In contrast, the IMUs were placed horizontally on the head and feet, which corresponded to their calibration axes. In addition, the IMU positioned over the trunk might have been tilted due to postural alignment, lumbar lordosis of the subject or small inaccuracies in the positioning of the IMUs [7]. However, a previous study [20] has shown that the error contribution is relatively small in comparison to the error caused by the anatomical frame discrepancy between the systems. The study quantified the accuracy of inertial sensors in a 3D anatomical joint angle measurement with respect to an instrumented gimbal. The gimbal rotated about three axes and directly measured the angles in the knee joint coordinate system recommended by the International Society of Biomechanics. Through the use of sensor attachment devices physically fixed to the gimbal, the joint angle estimation error, which occurs due to the inaccuracy of the sensor attachment matrix, was essentially eliminated, leaving only the error due to the inertial sensors. The angle estimation errors are smaller than those reported previously in human gait studies, which suggests that the sensor attachment may be a significant source of error in the inertial sensor measurements. These factors can be perceived as limitations of the estimations of the data from IMUs. It should be noted that the previous research has found that a significant challenge is the noise sources within the IMU sensors when compared to optical tracking systems, in particular during sporting activities, for example when measuring a dynamic range of a full-strength golf swing [10]. However, it has been shown that the analysis of kinematic parameters using a full-body IMU dataset can provide a reasonable accuracy compared with optical methods in alpine skiing [11].

In summary, the aim of this study was to validate the use of IMUs to measure axial movement parameters during turning. The ICCs showed excellent agreement for all axial movement parameters. Future studies could use a different method of synchronizing the IMUs with the Vicon data. This could be achieved by using a trigger signal to simultaneously start the system recording, although a lower technology solution could use sinusoidal rotations in the chair to map the time series of the signals. The latter has the advantage that the signals can be synchronized without additional software or hardware.

## 5. Conclusions

We can conclude that the level of agreement of IMUs with the gold standard camera-based systems indicates that these are appropriate for the assessment of turning behaviour and studying the effects of interventions. Although this current study considered healthy individuals, this shows the potential for the investigation of turning at different speeds with IMUs is feasible, which could be used in the assessment of individuals with neurological conditions. Indeed, the benefit of monitoring turning characteristics with small sensors that have low power requirements, such as IMUs, is that the clinician can determine axial segment behaviour, and the effectiveness of rehabilitation outside of the laboratory. Therefore, IMU sensors could be incorporated into rehabilitation strategies to measure turning characteristics especially in Parkinson’s disease patients, stroke patients and older adults. We predict that, in the near future, therapists will be able to use IMUs to evaluate the turning performance in neurological patients continuously during daily activities for a more ecological and realistic understanding of patients’ mobility, performance and falls risk. Physiotherapists may also be able to incorporate movement monitors to provide real-time feedback to improve motor learning and motor performance during treatment sessions. With technology becoming increasingly accessible and pervasive, therapists need to critically evaluate the advantages and limitations of implementing emerging technologies, such as IMUs, into their clinical practice. Therefore, mobility assessment in the home and community settings may provide important information for the clinician and patient to determine falls risk, disease progression, the effectiveness of rehabilitation and the potential benefits of preventative interventions.

## Figures and Tables

**Table 1:** Demonstrates the Coefficient of Determination (R^2^), the gradient (m) and the y intercept (c) of the linear regression best fit line comparing the displacement and velocity profiles of the two datasets.

Segments	Coefficient of Determination (R^2^)	Gradient (m)	Intercept (c)	*P*-Value
	Displacement (deg)			
**Head**	0.999	1.021	−0.078	<0.001
**Thorax**	0.983	0.978	3.009	<0.001
**Lt** **. Foot**	0.997	0.924	0.509	<0.001
**Rt** **. Foot**	0.992	0.954	1.226	<0.001
	**Velocity (deg/s)**			
**Head**	0.968	1.018	0.0732	<0.001
**Thorax**	0.777	0.685	15.589	<0.001
**Lt** **. Foot**	0.884	0.848	1.473	<0.001
**Rt** **. Foot**	0.856	0.805	3.010	<0.001

**Table 2:** The validity of Vicon motion analysis system and IMU measurement for all variables.

Variables	Vicon (Mean ± SD)	IMU (Mean ± SD)	ICC_(2,4__)_	95% CI	*P*-Value
Head reorientation onset (s)	0.56 ± 0.07	0.55 ± 0.08	0.87	0.80–0.92	0.01
Thorax reorientation onset (s)	0.59 ± 0.08	0.60 ± 0.09	0.82	0.75–0.87	0.046
Leading foot reorientation onset (s)	0.77 ± 0.14	0.79 ± 0.14	0.89	0.85–0.92	0.030
Trailing foot reorientation onset (s)	1.03 ± 0.25	0.99 ± 0.25	0.88	0.83–0.91	0.047
Head end time (s)	2.68 ± 0.66	2.66 ± 0.62	0.80	0.73–0.86	0.579
Thorax end time (s)	2.71 ± 0.68	2.66 ± 0.57	0.80	0.72–0.85	0.240
Peak head Yaw velocity (deg/s)	193.93 ± 76.54	197.13 ± 59.36	0.88	0.83–0.91	0.357
Peak head-thorax separation angle (deg)	13.17 ± 11.02	15.05 ± 11.17	0.83	0.76–0.81	0.040
Total steps (N)	4.04 ± 1.02	4.05 ± 0.98	0.97	0.96–0.98	0.639
Turn duration (s)	2.55 ± 0.71	2.54 ± 0.88	0.88	0.83–0.91	0.151
Step frequency (Hz)	2.32 ± 0.74	2.20 ± 0.83	0.80	0.73–0.85	0.141
Step size (deg)	68.93 ± 26.44	69.00 ± 25.72	0.88	0.90–0.96	0.888
